# Tequila Agave Bagasse Hydrolysate for the Production of Polyhydroxybutyrate by *Burkholderia sacchari*

**DOI:** 10.3390/bioengineering6040115

**Published:** 2019-12-17

**Authors:** Yolanda González-García, Janessa Grieve, Juan Carlos Meza-Contreras, Berenice Clifton-García, José Antonio Silva-Guzman

**Affiliations:** 1 Department of Wood, Cellulose and Paper, University of Guadalajara, 45020 Zapopan, Mexico; janessagrieve@me.com (J.G.); jcmezac@gmail.com (J.C.M.-C.); jasilva@dmcyp.cucei.udg.mx (J.A.S.-G.); 2 Department of Chemical Engineering, University of Guadalajara, 44430 Guadalajara, Mexico; bere_clifton@hotmail.com

**Keywords:** polyhydroxybutyrate, tequila bagasse, hydrolysate detoxification, activated charcoal, phenolic compounds

## Abstract

Tequila agave bagasse (TAB) is the fibrous waste from the Tequila production process. It is generated in large amounts and its disposal is an environmental problem. Its use as a source of fermentable sugars for biotechnological processes is of interest; thus, it was investigated for the production of polyhydroxybutyrate (PHB) by the xylose-assimilating bacteria *Burkholderia sacchari*. First, it was chemically hydrolyzed, yielding 20.6 g·L^−1^ of reducing sugars, with xylose and glucose as the main components (7:3 ratio). Next, the effect of hydrolysis by-products on *B. sacchari* growth was evaluated. Phenolic compounds showed the highest toxicity (> 60% of growth inhibition). Then, detoxification methods (resins, activated charcoal, laccases) were tested to remove the growth inhibitory compounds from the TAB hydrolysate (TABH). The highest removal percentage (92%) was achieved using activated charcoal (50 g·L^−1^, pH 2, 4 h). Finally, detoxified TABH was used as the carbon source for the production of PHB in a two-step batch culture, reaching a biomass production of 11.3 g·L^−1^ and a PHB accumulation of 24 g PHB g^−1^ dry cell (after 122 h of culture). The polymer structure resulted in a homopolymer of 3-hydroxybutyric acid. It is concluded that the TAB could be hydrolyzed and valorized as a carbon source for producing PHB.

## 1. Introduction

Polyhydroxyalkanoate (PHA) is a family of biodegradable and biocompatible polymers produced by microorganisms. They have great potential as a substitute for petroleum-based plastics as packing material, disposable items, and biomedical devices [1]:

Polyhydroxybutyrate (PHB) is the main representative of these polymers. Nowadays, some manufacturers such as Metabolix (USA), Tianjin Green Bioscience Co., Ltd. (China), and Biocycle PHB Industrial S.A (Brazil) commercialize this biopolymer, which is produced from raw materials such as food crops and sugarcane [1]. Nevertheless, its production cost is 5 to 10 times higher than that of conventional plastics; in particular, the substrate cost for PHA production represents almost half of the production cost [2].

Thus, the use of low-cost substrates for producing PHAs is a matter of interest. Among various alternative substrates, lignocellulosic wastes have gained considerable attention since they could be hydrolyzed to yield sugars (i.e., glucose, xylose, and arabinose) for fermentation processes [3]. Examples of this type of waste, studied for PHA production include sugar cane bagasse, rice, wheat straw, and corn stover [4].

Tequila agave bagasse (TAB) is a residual fibrous material from the Tequila production process. In 2018, 346,700.00 tons of TAB were generated. This waste did not have any specific use, but since it contains large amounts of cellulose and hemicellulose [5], it might be used as a low-cost substrate for fermentation processes. It has been hydrolyzed and used as a carbon source in submerged fermentation for the production of bioethanol, organic acids, and lipids [6,7]. Nevertheless, the research about the use of TAB for the production of PHAs is scarce [8] as well as the effect of the hydrolysis byproducts generated from its chemical saccharification on the growth and PHB accumulation by any PHA-producing microorganism. 

Lignocellulosic wastes conversion to PHAs generally requires a hydrolysis step to obtain fermentable sugars and then a detoxification process to remove inhibitory compounds produced during hydrolysis [9]. Different detoxification methodologies (activated charcoal, ionic resins, and enzymes) have been studied in bagasse hydrolysates, such as sugar cane bagasse hydrolysate, but there are not reports about their use for detoxification of TABH.

This research aimed to evaluate the effect of growth-inhibition compounds (present in the TABH) on the growth of the xylose-utilizing bacteria *B. sacchari*, their removal using activated charcoal, resins, and laccases, and the use of the detoxified TABH for the production of PHB.

## 2. Materials and Methods 

### 2.1. Characterization TAB

TAB fibers were washed (Sprout-Waldron refiner, D2A509NH) and then centrifuged to remove water, sun-dried for 48 h, ground, and sieved (60 mesh). Cellulose, hemicellulose, lignin, ashes, extractable, and humidity content were determined by the Technical Association of the Pulp and Paper Industry (TAPPI) standards (T203, T222, and T257).

### 2.2. Chemical Hydrolysis of TAB and Hydrolysate Characterization

Acid hydrolysis of TAB fibers was performed in 250 mL capped flasks using the following conditions: H_2_SO_4_, 2.3% (*w*/*v*); TAB: acid solution, 1:10 (*w*/*v*); temperature, 130 °C (autoclave); time, 20 min. Sixty mesh sieved TAB (250 μm fiber size), and not sieved TAB (mixed fiber sizes, from 125–420 μm) were used. After hydrolysis, the remaining fibers were separated by filtration (filter paper Whatman 2). Next, pH was adjusted to 5.5 by the addition of Ca(OH)_2_ (constant agitation), and the precipitated solids were removed by filtration (filter paper Whatman 2). The TABH was analyzed for determining: total sugars, reducing sugars, and total phenolic compounds (Phenol-sulfuric [10], DNS (dinitrosalicylic acid) [11], and Folin–Ciocalteu method [12], respectively). Monomeric sugars were analyzed by HPLC (Waters) with an IR detector and using an Aminex 87P column: mobile phase, water; flow 0.6 mL min^−1^; temperature, 80 °C; sample volume, 20 μL).

### 2.3. Microorganism and Culture Media

The strain *B. sacchari* 17165 was purchased from DSMZ, reactivated according to DSMZ instructions in R2A medium (in g·L^−1^): yeast extract, 0.5; meat peptone 0.5; casein peptone, 0.5; glucose, 0.5; soluble starch, 0.5; sodium pyruvate, 0.3; K_2_HPO_4_, 0.3; and MgSO_4_·7H_2_O, 0.05; pH 7. The strain was incubated at 30 °C and 150 rpm for 48 h and then frozen at −20 °C (2 mL microtubes with 0.8 mL of bacterial suspension and 0.2 mL of glycerol).

Two culture media were studied for *B. sacchari* growth and PHB production: control medium and Detoxified TABH medium. Control medium composition was (in g·L^−1^): xylose, 14; glucose, 6; KH_2_PO_4_, 2.5; Na_2_HPO_4_, 5.5; (NH_4_)_2_SO_4_, 6; MgSO_4_·7H_2_O, 0.5; CaCl_2_·2H_2_O, 0.02; ammonium ferric citrate, 0.1; peptone, 1; and yeast extract, 1. Trace elements solution (1 mL·L^−1^) with the following composition (g·L^−1^) was also added: H_3_BO_3_, 0.30; CoCl_2_·6H_2_O, 0.20; ZnSO_4_·7H_2_O, 0.1; MnCl_2_·4H_2_O, 0.03; NaMoO_4_·2H_2_O, 0.03; NiCl_2_·6H_2_O, 0.02; and CuSO_4_·5H_2_O, 0.01. 

The Detoxified TABH medium was based on the hydrolysate (20 g·L^−1^ of reducing sugars, xylose: glucose ratio of 7:3) with the addition of all the components above mentioned except for the sugars.

pH was adjusted to 6.5, and culture media were autoclaved (peptone and yeast extract were autoclaved separately) before inoculation with 10% (*v*/*v*) of *B. sacchari* pre-culture (OD_600nm_ = 0.5). The incubation conditions used were 30 °C and 150 rpm.

### 2.4. B. sacchari Growth Inhibition by TABH 

TABH was used as a culture medium, without diluting and diluted with water (5 times), in order to determine the microorganism’s tolerance to it. The sugars content of the diluted TABH were adjusted to the same concentration and proportion of the concentrated TABH, and the mineral salts, peptone, and yeast extract (as mentioned in Materials and Methods section) were added to both hydrolysates. After sterilization, 125 mL glass vials media (22 mL of TABH) were inoculated with 3 mL of *B. sacchari* pre-culture (control medium, OD_600nm_ = 0.5) and incubated under the conditions previously mentioned. 

Microbial growth (Dry cell mass) at 0, 24, 48, and 72 h was estimated by measuring the optical density of the sample at 600 nm and by its correlation with the dry cell weight, obtained gravimetrically.

### 2.5. B. sacchari Growth Inhibition by Model Toxic Compounds 

Acid hydrolysis by-products reported in other hydrolysates (inhibitory compounds) were added separately to the control medium (concentrated solutions previously sterilized), in order to evaluate their particular effect on *B. sacchari* growth. The following compounds (0.5–12 g·L^−1^) were studied: furfural, hydroxymethylfurfural, vanillin, coumaric acid, and ferulic acid. Glass vials of 125 mL containing 22 mL of control medium (added with each inhibitory compound) were inoculated with 3 mL of *B. sacchari* pre-culture (control medium, OD_600nm_ = 0.5), and the medium without inhibitory compounds was used as control. 

Microbial growth was measured as previously mentioned at 0, 24, 48, and 72 h, and maximum biomass production was used for determining growth inhibition percentage I% as follows:

I = (X_max_C − X_max_I) × 100 / X_max_C

where,

X_max_C = maximum biomass produced in the control medium,

X_max_I = maximum biomass produced in the control medium with the inhibitory compound.

### 2.6. Detoxification of TABH by Different Methods

Elimination of growth inhibitory compounds, specifically total phenolic compounds, from the TABH (without diluting) was studied using activated charcoal (Golden bell) and resins (Sigma & Aldrich): DOWEX 1 × 8 (chloride form); DOWEX G26 (hydrogen form); Amberlite XAD7 (hydrophobic). Before use, resins were washed with distilled water (agitation, 5 min) and activated according to manufacturer indications (agitation, 1 h): DOWEX G26, HCl 0.4 N; DOWEX 1 × 8 NaOH 0.1 N; Amberlite XAD7, methanol. Finally, resins were washed three times with distilled water [13].

Each adsorbent (50 mg·mL^−1^) was added separately into 10 mL glass vials with 2 mL of TABH at pH 2 or 7 (previously adjusted with NaOH or H_2_SO_4_) and kept in agitation (150 rpm) in a shaker at 25 °C (1 and 4 h). Next, the adsorbent was separated by filtration, and the total phenolic compounds were quantified by the Folin–Ciocalteu method. The phenolic compound elimination percentage E% was calculated as follows:

E = (P_t0_ − P_tf_) × 100 / P_t0_

where,

P_t0_ = Total phenolic compound before the detoxification process,

Ptf = Total phenolic compound after the detoxification process.

Detoxification of TABH using enzymes (*Trametes versicolor* laccase, Sigma & Aldrich 38429) was also studied. The pH was first adjusted to 5.5, and the effect of enzyme concentration (1 and 10 U) and contact time (1 and 5 h) on phenolic compounds elimination was investigated (2 mL microtubes, 50 °C, 150 rpm). 

### 2.7. PHB Production from Detoxified TABH by B. sacchari

The detoxified TABH medium and the control medium were used for the production of PHB by *B. sacchari* in 500 mL Erlenmeyer flasks containing 90 mL of culture medium, and inoculated with 10 mL of bacterial pre-culture (DO_600nm_ = 0.5, at 30 °C, and 150 rpm).

A two-step batch culture was performed as follows: (1) Biomass production (72 h) in medium (TABH or control) with nitrogen sources ((NH_4_)_2_SO_4,_ peptone and yeast extract) as described in the Materials and Methods Section; (2) PHB accumulation (48 h) in nitrogen-limited medium (TABH or control with 0.6 g·L^−1^ of (NH_4_)_2_SO_4_, without either peptone or yeast extract). For PHB accumulation, cells from the biomass production step were aseptically recovered by centrifugation and resuspended in nitrogen-limited culture media. Samples (5 mL) were withdrawn periodically and analyzed for quantifying biomass (dry cell weight), reducing sugars (DNS), pH, and PHB (dry weight) after solvent extraction. For polymer extraction, biomass was lyophilized, weighted, suspended in ethanol, and kept in constant agitation (24 h). Next, it was air-dried, suspended in chloroform, and kept in constant agitation (24 h). Cell debris was removed by filtration, and the organic phase was added to cold methanol (1:10 *v*/*v*) and kept in the freezer for 24 h for PHB precipitation. The polymer was recovered by centrifugation, air-dried, and weighed.

### 2.8. PHB Characterization

The polymer extracted from *B. sacchari* cells was analyzed by ATR-FTIR (Fourier Transform Infrared-Attenuated Total Reflectance) using a Perkin Elmer Spectrum Two FTIR spectrometer. For determining its monomeric composition, the polymer was subjected to acid methanolysis [14]. The resulting methyl esters were analyzed using a Perkin Elmer XL gas chromatograph, equipped with a CP-Wax 52 CB capillary column (25 m × 0.32 mm) and a flame ionization detector. The chromatographic conditions used were: sample injection volume, 1 μL; gas carrier, nitrogen; flow rate, 20 cm·s^−1^; injector and detector temperatures, 210 and 220 °C, respectively. A temperature ramp was used as follows: 50 °C for 1 min, incrementing by 8 °C min^−1^, and 160 °C for 5 min. Methyl benzoate and polyhydroxybutyrate from Fluka (after methanolysis) were used as internal and external standards, respectively.

## 3. Results and Discussions

### 3.1. Characterization of TAB

To know the cellulose, hemicellulose, lignin, extractable, ashes, and humidity content of the TAB to be used, it was characterized by TAPPI standards. Table 1 presents the data obtained, as expected, cellulose was found as the primary polymer followed by hemicellulose and lignin. Compared to other TAB characterizations previously reported, there was no significant variation in respect to the proportions of the structural components of the bagasse used in this research: cellulose, 20–50%; hemicellulose, 19–27%; and lignin, 15–20% [5,15].

### 3.2. Chemical Hydrolysis of TAB 

The effect of the TAB fiber size on the concentration of total and reducing sugars, xylose, glucose, and total phenolic compounds was investigated. The results are presented in Table 2. A slightly higher concentration of reducing sugars was obtained from the mixed TAB fiber sizes (20.6 vs. 19.14 g·L^−1^), but generally, the effect of the fiber size on the hydrolysates composition was not significant. 

The factors that usually influence the effectivity of the acid hydrolysis of lignocellulosic materials are the temperature and time of the process [16]. In some investigations, the influence of the fiber size had also been studied [17]. The benefit of grinding the fibers before performing the chemical hydrolysis is to increase the exposure area of the lignocellulosic material to the acid, as well as to reduce the crystallinity of cellulose, allowing it to hydrolyze with little generation of total phenolic compounds. Nevertheless, since sieving (60 mesh) the ground TAB to obtain a particular fiber size (250 μm) did not had a significant impact in the amount of reducing sugar or phenolic compounds obtained, this step could be avoided, representing a saving in time and energy. 

TAB hydrolysis under similar conditions to those used in this investigation was reported previously [5], and a concentration of 24.9 g·L^−1^ of reducing sugars was achieved (Table 3). Similar results obtained in hydrolysis studies of different materials are presented in Table 3.

The determination of monomeric sugars was performed using HPLC, finding that the xylose to glucose ratio was around 7:3. Xylose was expected to be found in higher concentrations than glucose since xylose is the main product of the hydrolysis of hemicellulose. Hemicellulose, because of its branched chemical structure, results in being more easily hydrolyzed in comparison to cellulose, whose principal degradation product is glucose. The high crystallinity of cellulose makes it challenging to hydrolyze into monomers of glucose [22]. This tendency has been reported in other investigations using sugar cane bagasse [23,24] and wheat straw [25]. Generally, the high percentage of xylose present in the TABH would be a negative aspect, given that it is not an easily assimilated substrate for the majority of the PHA-producing microorganisms. However, *B. sacchari* is capable of metabolizing xylose [23] therefore, this should not be a limiting factor for the growth and synthesis of PHB by it. 

### 3.3. B. sacchari Growth Inhibition by Toxic Compounds Present in the TABH

**Figure 1:**
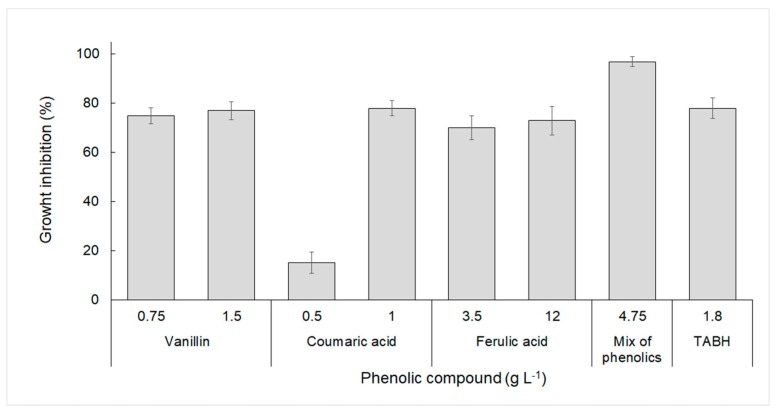
Inhibition of *B. sacchari* growth by model phenolic compounds associated with the acid hydrolysis of lignocellulosic materials.

TABH was used as a culture medium, concentrated and diluted with water (5 times), in order to determine the microorganism’s tolerance to it. It was found that only the diluted hydrolysate supported growth. On the other hand, the regular hydrolysate strongly inhibited the growth of *B. sacchari* (Figure 1), which evidences its toxic nature due to the presence of inhibitory compounds from the acid hydrolysis process.

The effect of pure inhibitory compounds such as furfural, hydroxymethylfurfural, acetic acid, levulinic acid, and phenolic compounds (vanillin, coumaric, and ferulic acids) on *B. sacchari* growth was investigated. It was found that the phenolic compounds caused the highest growth inhibition (above 60%), rather than the other inhibitory compounds investigated. The inhibitory effect of phenolic compounds (lowest and higher concentration used) are presented in Figure 1. Except for coumaric acid at 0.5 g·L^−1^, all the phenolic compounds tested strongly inhibited *B. sacchari* growth, while their mixture was even highly inhibitory (97% inhibition). Such toxic synergistic behavior has been previously described for other bacteria [26]. 

As previously mentioned, various types of bagasse hydrolysates presented concentrations of total phenolic compounds ranging from 0.13 g·L^−1^ to 3 g·L^−1^ with different levels of toxicity to the microorganism used [27,28]. The amount of total phenolic compounds produced during acid hydrolysis of the TAB was significant (1.6–1.7 g·L^−1^). Such compounds are known for decreasing the microbial growth rate associated with the loss of integrity of the cell membranes [26].

The evident growth inhibition of *B. sacchari* by the TABH confirmed the necessity of a detoxification treatment before using it as a cultivation media.

### 3.4. Elimination of Growth Inhibitory Compounds from TABH

**Figure 2:**
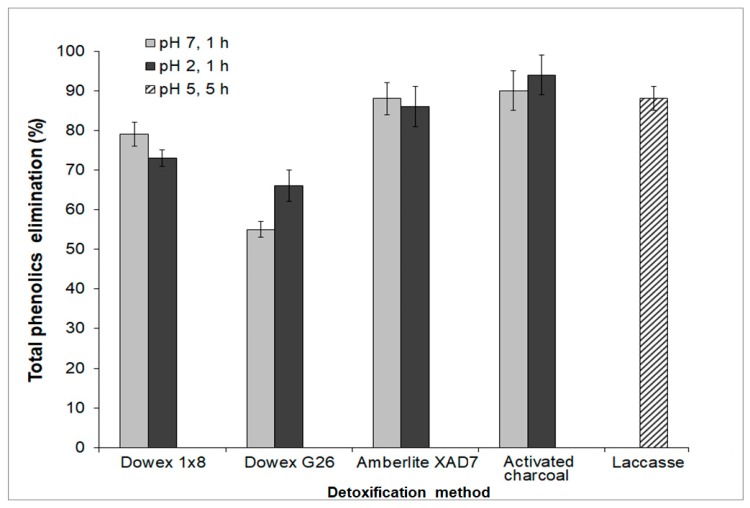
Total phenolic compounds removed from the TABH by different detoxification methods. For the treatment with resins and activated charcoal, 50 mg of adsorbent per mL of TABH were used. For laccase treatment, 1 U·mL^−1^ was used.

Different detoxification methods were used in order to eliminate growth inhibitors (with emphasis on phenolic compounds) from the TABH, and the results are presented in Figure 2.

The detoxification with activated charcoal is a treatment frequently used to purify or to recover certain compounds from hydrolysates (lignin, tannin, furan derivatives, aromatic monomers, and phenolic acids) [29,30]. The pH of the hydrolysate, the concentration of activated carbon used, and the contact time are known factors that can influence the effectivity of the detoxification process with this adsorbent [31,32]. The phenolics elimination percentage using this method was around 90%, which is similar to values reported for other hydrolysates (sugarcane bagasse, 94%; olive tree pruning residue, 98%) [23,31]. The adsorptive behavior observed for the phenolic compounds on activated carbon could be explained by their polarity (electron distribution), hydrophobicity, and chemical structure [33]. The pH can affect its absorption capacity because it can influence the nature of functional groups for both adsorbent and adsorbate. Weak organic acids and phenolic compounds are better absorbed when they are in a non-ionized state (pH < 4) [34], this could be a possible explanation for the slight increase in the elimination of phenolic compounds at pH 2. 

Regarding the elimination of phenolic compounds from TABH by the resins, it was from 55% to 88%; this result is similar to other values reported in the literature (64–94%) [35,36,37,38]. Using the Dowex 1 × 8 resin at acid pH, the amount of phenolic compounds adsorbed was lower than at pH 7; a comparable result was observed by Martos et al. [39]. However, the elimination of phenolic compounds using this adsorbent was higher (73–78%) than the achieved with the cation-exchange resin Dowex G26 (55–65%), which might be less efficient to adsorb phenolic compounds due to its overall negative charge. The same behavior was observed previously [36] using a similar resin (AG 50W-X8). Concerning the use of the Amberlite XAD7 resin, the elimination percentages of phenolic compounds were high (86–88%). This resin is an acrylic ester that is slightly polar and has been used for eliminating phenolic compounds from olive mill wastewater [40]. 

The last method assayed for detoxifying the TABH was the use of laccases. The elimination of total phenolic compounds by this method was 88%. The action mechanism of the laccase enzyme has been previously elucidated. The enzyme oxidizes phenolic compounds that lead to the formation of phenoxy-type free radicals, which are unstable and polymerize into aromatic compounds that are less toxic [41]. Other compounds present in the hydrolysate (such as salts) might inhibit to a certain extent the enzyme activity. This is a probable reason why the elimination of phenolic compounds did not reach above 90% [42]. Similar results (70–75% of phenolic compounds elimination) were obtained using laccases for detoxifying wheat straw hydrolysate (0.5 U·mL^−1^ of laccase at pH 5 for 2 h) [42].

### 3.5. B. sacchari Growth in TABH Detoxified by Different Methods

**Figure 3:**
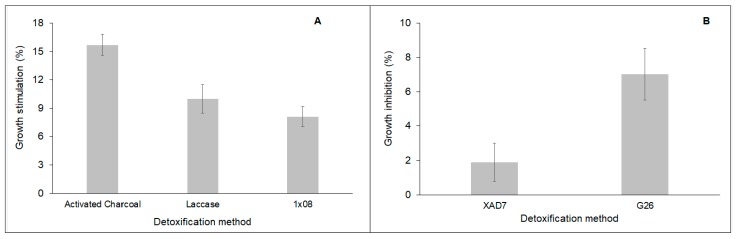
*B. sacchari* growth stimulation (**A**) or inhibition (**B**) by TABH detoxified using different methods.

*B. sacchari* growth was evaluated in the detoxified TAB hydrolysates, and it was found that some hydrolysates stimulated the growth, with respect to the control medium (Figure 3A), while others were still inhibitory (Figure 3B). The TABH treated with activated charcoal allowed the highest biomass production, 16% more biomass (Figure 3A) than the presented in the control medium, followed by the TABH detoxified with enzymes (10% more biomass than the produced in the control medium). Concerning the TABH treated with resins, the growth of *B. sacchari* with respect to the control medium varied: it was slightly inhibited in TABH detoxified with XAD7 or G26 resin (3 and 7% less biomass, respectively) (Figure 3B), but enhanced (8%) in TABH treated with 1 × 8 resin (Figure 3A). 

Thus, the TABH treated with activated charcoal, resin 1 × 8, and enzymes not only supported the growth of *B. sacchari* but stimulated it. It is possible that such detoxification methods removed toxic compounds but left tolerable concentrations of some others that could act as growth factors, an effect that has been previously observed [26]. It has been mentioned that some organic acids and phenol-type compounds (including formic, acetic, levulinic, 4-hydroxybenzoic and gallic, and vanillic acid) at low concentration (below 10 mM) could stimulate the cell growth instead of suppressing it [43,44].

On the other hand, detoxification of TABH with XAD7 and G26 resins might not absorb other known toxic compounds such as furfural and HMF, which acted as growth inhibitors [35].

Activated charcoal eliminated more than 90% of total phenolic compounds from the TABH and the detoxified TABH not only supported but promoted the growth of *B. sacchari*, thus this treatment (50 g·L^−1^, pH 2, and a contact time of 4 h) was selected for detoxifying the hydrolysate used in the PHB production experiment. 

### 3.6. PHB Production from TABH Detoxified with Activated Charcoal

**Figure 4:**
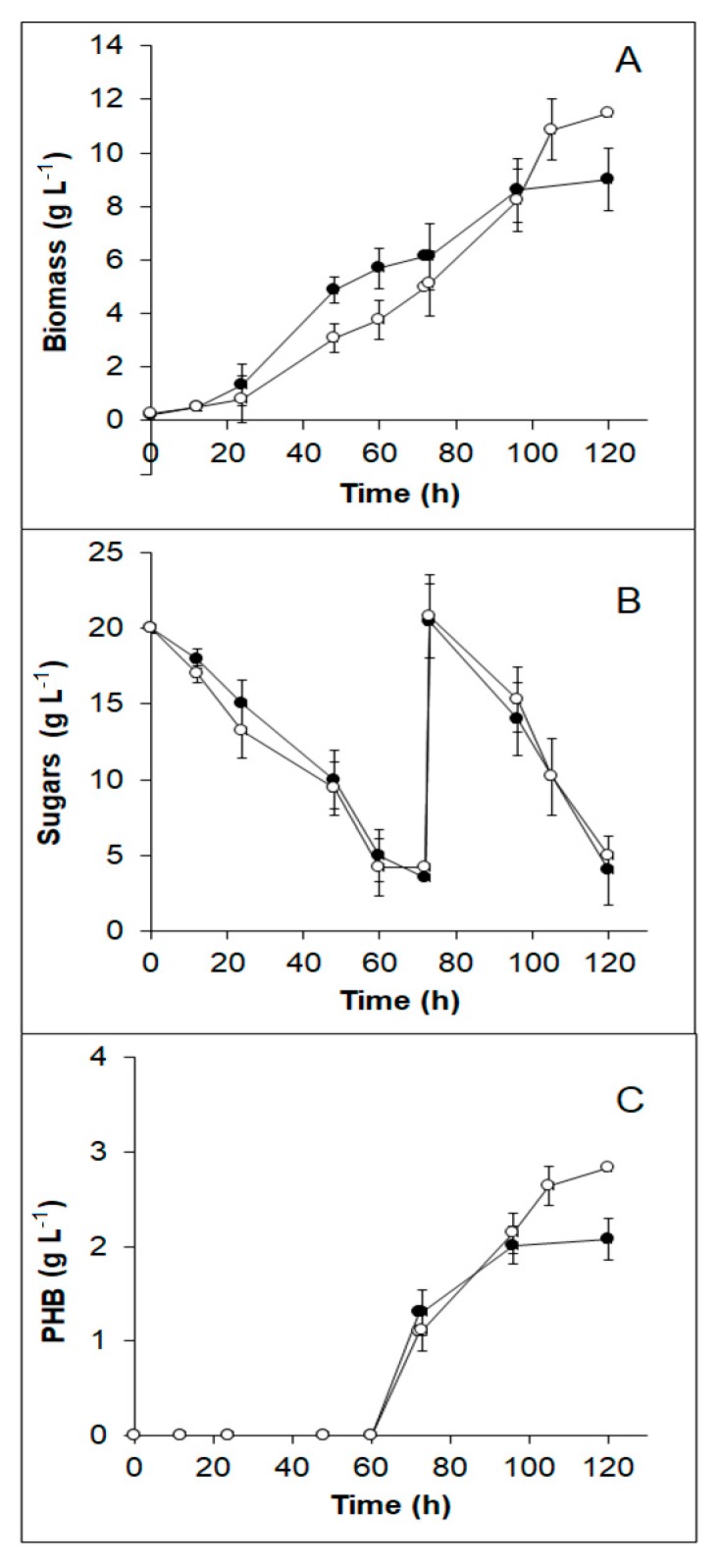
Kinetic profile of *B. sacchari* growing in TABH detoxified with activated charcoal (○) and control medium (●). (**A**) Biomass production. (**B**) Substrate consumption. (**C**) Polyhydroxybutyrate (PHB) production.

The production of PHB using *B. sacchari* from TABH detoxified with activated carbon, was investigated. After detoxification, TABH pH was adjusted to 7. Synthetic media with the same sugar concentration (20 g·L^−1^) and xylose to glucose proportion (7:3) as the TABH was used as a control medium. Kinetics of *B. sacchari* growth, sugars consumption, and PHB production are depicted in Figure 4.

In both media, the lag phase was not evident, while exponential growth occurred from 12 to 72 h. Diauxic growth due to the presence of at least two carbon sources (xylose and glucose) was almost imperceptible, although a slight shift in the growth curve is observed at 40 h in both media used. At 72 h cells were harvested and resuspended in nitrogen-limited medium, cell growth continued until 96 h (control medium), and 105 h (TABH medium), although it represents both the PHB and non-PHB biomass (residual biomass). This is consistent with the PHB accumulation profile (Figure 4C) since the polymer production occurred from 72 to 96 h in the control medium, and from 72 to 105 h in TABH medium. The sugar consumption profile was similar in both culture media (Figure 4B).

The results of the kinetic parameters are presented in Table 4. Biomass and PHB production, as well as the maximum growth rate and PHB accumulation, were slightly higher in TABH medium (1.05–1.2 times) than in the control medium. Such results have been observed in other PHA producing bacteria growing in hydrolysates, and it has been hypothesized and researched that certain phenolic compounds and organic acids present in the hydrolysates in minimal concentrations, can stimulate growth and production of PHB [23]. 

The PHB accumulation percentage achieved by *B. sacchari* from TABH, compares with values that have been reported from other hydrolysates (in shake flasks) by different strains: 34%, *Halomonas boliviensis* (wheat bran) [45]; 31.9% of PHB, *Ralstonia eutropha* (pulp fiber sludge); and 32% of PHB, *Sphingobium scionense* (softwood) [9].

*B. sacchari* has been previously used for PHB production from other lignocellulosic materials hydrolysates. The results of biomass production and PHB accumulation obtained from TABH are similar to those values reported from sugar cane bagasse hydrolysate (shaken flasks) by the same strain: 6.13 g·L^−1^, and 23.22%, respectively [23].

The biomass and PHB yields (on the substrate) obtained in the control medium and in the TABH medium were similar (0.23 and 0.10 g·g^−1^, respectively). Specifically, Y_P/S_ value obtained for *B. sacchari* growing in the TABH medium is low compared to those reported for other hydrolysates (0.11 to 0.46 g·g^−1^) [23].

Although flask fermentations are very useful to study fermentation processes, they are restricted due to the incapacity to be controlling variables such as pH and dissolved oxygen. These are essential factors to optimize microbial growth and PHB accumulation. During the flask fermentation, the pH dropped from 7 to 5 and therefore affected the accumulation of the biopolymer [23]; thus, the production of PHB from TABH could be further optimized by using an automatized bioreactor and implementing a fed-batch system.

### 3.7. PHB Characterization

**Figure 5:**
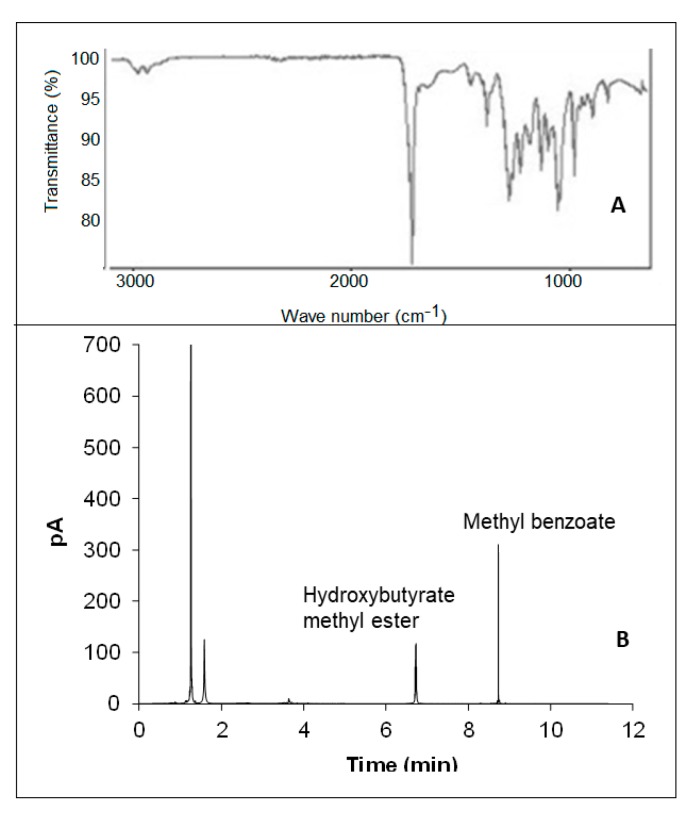
Characterization of the PHB produced by *B. sacchari* from TABH detoxified with activated charcoal. FTIR spectrum (**A**); GC chromatogram (**B**).

An FTIR analysis was performed on the polymer produced from detoxified TABH (Figure 5A), and it was compared against a Fluka ™ PHB standard. The peak around 2900 cm^−1^ is characteristic of carbon to hydrogen bonds, which are a part of the general structure of PHAs. The zone between 1700 and 1750 cm^−1^ relates to the stretching carbonyl C=O group, and the set of peaks from 1300 to 1000 cm^−1^ to the stretching of C–O bonds, both signals correspond to the ester bonds present in the PHAs structure. The peak around 1450 cm^−1^ originates from the asymmetric deformation and stretching of the bonds of methyl groups C–H, the same as the peak approximately at 1380 cm^−1^ [46]. 

The monomeric composition of the synthesized PHA was investigated by gas chromatography (GC). As depicted in Figure 5B, the biopolymer produced by *B. sacchari*, using the TABH as carbon source, is a homopolymer of 3-hydroxybutyric acid.

In previous experiments with *B. sacchari* using mixtures of glucose and xylose, as well as hydrolysates (wheat straw, sugar cane bagasse) as carbon sources, the polymer produced was also composed of repeating units of 3-hydroxybutyric acid [23,47]. 

## Figures and Tables

**Table 1:** TAB characterization.

Component	Content (%)
Cellulose	50.1 ± 2.1
Hemicellulose	21.1 ± 2.4
Lignin	13.1 ± 1.3
Extractable	8.0 ± 1.1
Ashes	0.8 ± 0.1
Humidity	7.0 ± 0.9

**Table 2:** Tequila agave bagasse hydrolysate (TABH) composition after acid hydrolysis of different Tequila agave bagasse (TAB) fiber size.

Compound	Fiber Size	
	Mixed (125–420 μm)	60 mesh (250 μm)
Total sugars (g·L^−1^)	25.5 ± 1.5	23.9 ± 1.9
Reducing sugars (g·L^−1^)	20.61 ± 0.92	19.14 ± 1.03
Xylose (%) ^1^	72	71
Glucose (%)	28	29
Total phenolic compounds (g·L^−1^)	1.7 ± 0.12	1.6 ± 0.13

^1^ Percentage concerning the total amount of reducing sugars.

**Table 3:** Chemical composition of hydrolysates obtained from different lignocellulosic materials.

Lignocellulosic Material	Reducing Sugars (g·L^−1^)	Phenolic Compounds (g·L^−1^)	Reference
TAB	24.9	n.r.	[5]
Sugarcane bagasse	25.38	n.r.	[18]
Sugarcane bagasse	n.r.	2.86	[19]
Sago trunk cortex	29.46	2.15	[20]
Sugarcane bagasse	30.29	2.75	[13]
*Saccharum spontaneum*	32.15	2.01	[21]

n.r.—not reported.

**Table 4:** Biomass and PHB production by *B. sacchari* from activated charcoal detoxified TABH and mineral medium (120 h).

Parameter	Control Medium (CM)	TABH (Detoxified)
Total biomass (g·L^−1^)	8.78 ± 1.04	11.03 ± 1.14
Residual biomass (g·L^−1^) ^a^	6.77 ± 1.09	8.36 ± 0.91
PHB (g·L^−1^)	2.01 ± 0.86	2.67 ± 0.96
PHB (%) ^b^	22.91 ± 1.18	24.20 ± 1.26
μ_max_ (h^−1^)	0.08 ± 0.01	0.11 ± 0.02
Y_X/S_ (g·g^−1^) ^c^	0.23 ± 0.02	0.25 ± 0.02
Y_P/S_ (g·g^−1^) ^d^	0.10 ± 0.01	0.10 ± 0.01

^a^ Total Biomass—PHB. ^b^ g of PHB g^−1^ total biomass × 100. ^c^ g of residual biomass g^−1^ reducing sugar consumed. ^d^ g of PHB g^−1^ reducing sugar consumed.

## References

[1] Tsang Y.F., Kumar V., Samadar P., Yang Y., Lee J., Ok Y.S., Song H., Kim K.-H., Kwon E.E., Jeon Y.J. (2019). Production of bioplastic through food waste valorization. Environ. Int..

[2] Salgaonkar B.B., Bragança J.M. (2017). Utilization of Sugarcane Bagasse by *Halogeometricum borinquense* Strain E3 for Biosynthesis of Poly(3-hydroxybutyrate-co-3-hydroxyvalerate). Bioengineering.

[3] Sandhya M., Aravind J., Kanmani P. (2012). Production of polyhydroxyalkanoates from *Ralstonia eutropha* using paddy straw as cheap substrate. Int. J. Environ. Sci. Technol..

[4] Nielsen C., Rahman A., Rehman A.U., Walsh M.K., Miller C.D. (2017). Food waste conversion to microbial polyhydroxyalkanoates. Microb. Biotechnol..

[5] Saucedo-Luna J., Castro-Montoya A.J., Rico J.L., Campos-García J. (2010). Optimization of acid hydrolysis of bagasse from *Agave tequilana* Weber. Rev. Mex. Ing. Quim..

[6] Aguilar D.L., Rodríguez-Jasso R.M., Zanuso E., de Rodríguez D.J., Amaya-Delgado L., Sanchez A., Ruiz H.A. (2018). Scale-up and evaluation of hydrothermal pretreatment in isothermal and non-isothermal regimen for bioethanol production using agave bagasse. Bioresour. Technol..

[7] Niehus X., Crutz-Le Coq A.-M., Sandoval G., Nicaud J.-M., Ledesma-Amaro R. (2018). Engineering *Yarrowia lipolytica* to enhance lipid production from lignocellulosic materials. Biotechnol. Biofuels.

[8] Alva Munoz L.E., Riley M.R. (2008). Utilization of cellulosic waste from tequila bagasse and production of polyhydroxyalkanoate (PHA) bioplastics by *Saccharophagus degradans*. Biotechnol. Bioeng..

[9] Obruca S., Benesova P., Marsalek L., Marova I. (2015). Use of lignocellulosic materials for PHA production. Chem. Biochem. Eng. Q..

[10] Dubois M., Gilles K., Hamilton J., Rebers P., Smith F. (1956). Colorimetric method based on phenol sulfuric acid. Anal. Chem..

[11] Miller G.L. (1956). Use of dinitrosalicylic acid reagent for determination of reducing sugar. Anal. Chem..

[12] Singleton V.L., Orthofer R., Lamuela-Raventos R.M. (1999). Analysis of total phenols and other oxidation substrates and antioxidants by means of Folin–Ciocalteau reagent. Method. Enzymol..

[13] Chandel A.K., Kapoor R.K., Singh A., Kuhad R.C. (2007). Detoxification of sugarcane bagasse hydrolysate improves ethanol production by *Candida shehatae* NCIM 3501. Bioresour. Technol..

[14] Linton E., Rahman A., Viamajala S., Sims R.C., Miller C.D. (2012). Polyhydroxyalkanoate quantification in organic wastes and pure cultures using a single-step extraction and 1H NMR analysis. Water Sci. Technol..

[15] Núñez H.M., Rodríguez L.F., Khanna M. (2011). Agave for tequila and biofuels: An economic assessment and potential opportunities. GCB Bioenergy.

[16] Mussatto S.I., Roberto I.C. (2004). Alternatives for detoxification of diluted-acid lignocellulosic hydrolyzates for use in fermentative processes: A review. Bioresour. Technol..

[17] Zamudio-Jaramillo M.A., Castro-Montoya A.J., Yescas R.M., Parga M.D.C.C., Hernández J.C.G., Luna J.S. (2014). Optimization of particle size for hydrolysis of pine wood polysaccharides and its impact on milling energy. IJRER.

[18] Laopaiboon P., Thani A., Leelavatcharamas V., Laopaiboon L. (2010). Acid hydrolysis of sugarcane bagasse for lactic acid production. Bioresour. Technol..

[19] Martinez A., Rodriguez M.E., Wells M.L., York S.W., Preston J.F., Ingram L.O. (2001). Detoxification of dilute acid hydrolysates of lignocellulose with lime. Biotechnol. Prog..

[20] Kamal S.M.M., Mohamad N.L., Abdullah A.G.L., Abdullah N. (2011). Detoxification of sago trunk hydrolysate using activated charcoal for xylitol production. Procedia Food Sci..

[21] Chandel A.K., Singh O.V., Rao L.V., Chandrasekhar G., Narasu M.L. (2011). Bioconversion of novel substrate *Saccharum spontaneum*, a weedy material, into ethanol by *Pichia stipitis* NCIM3498. Bioresour. Technol..

[22] Palmqvist E., Hahn-Hägerdal B. (2000). Fermentation of lignocellulosic hydrolysates. I: Inhibition and detoxification. Bioresour. Technol..

[23] Silva L., Taciro M., Ramos M., Carter J., Pradella J., Gomez G. (2004). Poly-3-hydroxybutyrate (P3HB) production by bacteria from xylose, glucose, and sugarcane bagasse hydrolysate. J. Ind. Microbiol. Biot..

[24] Liu R., Liang L., Cao W., Mingke W., Chen K., Ma J., Jiang M., Wei P., Ouyang P. (2012). Succinate production by metabolically engineered *Escherichia coli* using sugarcane bagasse hydrolysate as the carbon source. Bioresour. Technol..

[25] Nigam J.N. (2001). Ethanol production from wheat straw hemicellulose hydrolysate by *Pichia stipitis*. J. Biotechnol..

[26] Palmqvist E., Hahn-Hägerdal B. (2000). Fermentation of lignocellulosic hydrolysates. II: Inhibitors and mechanisms of inhibition. Bioresour. Technol..

[27] Millati R., Niklasson C., Taherzadeh M.J. (2002). Effect of pH, time and temperature of overliming on detoxification of dilute-acid hydrolyzates for fermentation by *Saccharomyces cerevisiae*. Proc. Biochem..

[28] Monlau F., Sambusiti C., Barakat A., Quéméneur M., Trably E., Steyer J.-P., Carrere H. (2014). Do furanic and phenolic compounds of lignocellulosic and algae biomass hydrolyzate inhibit anaerobic mixed cultures? A comprehensive review. Biotechnol. Adv..

[29] Liu X., Fatehi P., Ni Y. (2012). Removal of inhibitors from pre-hydrolysis liquor of kraft-based dissolving pulp production process using adsorption and flocculation processes. Bioresour. Technol..

[30] Zhang Y., Xia C., Lu M., Tu M. (2018). Effect of overliming and activated carbon detoxification on inhibitors removal and butanol fermentation of poplar prehydrolysates. Biotechnol. Biofuels..

[31] Mateo S., Roberto I.C., Sánchez S., Moya A.J. (2013). Detoxification of hemicellulosic hydrolyzate from olive tree pruning residue. Ind. Crop. Prod..

[32] Sarawan C., Suinyuy T., Sewsynker-Sukai Y., Kana E.B. (2019). Optimized activated charcoal detoxification of acid-pretreated lignocellulosic substrate and assessment for bioethanol production. Bioresour. Technol..

[33] Michailof C., Stavropoulos G.G., Panayiotou C. (2008). Enhanced adsorption of phenolic compounds, commonly encountered in olive mill wastewaters, on olive husk derived activated carbons. Bioresour. Technol..

[34] Costa T.D.S., Rogez H., Pena R.D.S. (2015). Adsorption capacity of phenolic compounds onto cellulose and xylan. Food Sci. Technol..

[35] Carvalheiro F., Duarte L.C., Lopes S., Parajó J.C., Pereira H., Gírio F.M. (2005). Evaluation of the detoxification of brewery’s spent grain hydrolysate for xylitol production by *Debaryomyces hansenii* CCMI 941. Proc. Biochem..

[36] Nilvebrant N.-O., Reimann A., Larsson S., Jönsson L.J. (2001). Detoxification of lignocellulose hydrolysates with ion-exchange resins. Appl. Biochem. Biotechnol..

[37] Mota M.I.F., Barbosa S., Pinto P.C.R., Ribeiro A.M., Ferreira A., Loureiro J.M., Rodrigues A.E. (2019). Adsorption of vanillic and syringic acids onto a macroporous polymeric resin and recovery with ethanol:water (90:10 %V/V) solution. Sep. Purif. Technol..

[38] Nitzsche R., Gröngröft A., Kraume M. (2019). Separation of lignin from beech wood hydrolysate using polymeric resins and zeolites—Determination and application of adsorption isotherms. Sep. Purif. Technol..

[39] Martos N., Sánchez A., Molina-Díaz A. (2005). Comparative study of the retention of nine phenolic compounds on anionic exchanger resins. Chem. Pap..

[40] Bertin L., Ferri F., Scoma A., Marchetti L., Fava F. (2011). Recovery of high added value natural polyphenols from actual olive mill wastewater through solid phase extraction. Chem. Eng. J..

[41] Moreno A.D., Ibarra D., Fernández J.L., Ballesteros M. (2012). Different laccase detoxification strategies for ethanol production from lignocellulosic biomass by the thermotolerant yeast *Kluyveromyces marxianus* CECT 10875. Bioresour. Technol..

[42] Jurado M., Prieto A., Martínez-Alcalá A., Martínez A.T., Martínez M.J. (2009). Laccase detoxification of steam-exploded wheat straw for second generation bioethanol. Bioresour. Technol..

[43] Huang C., Wu H., Liu Q., Li Y., Zong M. (2011). Effects of aldehydes on the growth and lipid accumulation of oleaginous yeast *Trichosporon fermentans*. J. Agric. Food Chem..

[44] Guo Z., Olsson L. (2014). Physiological response of Saccharomyces cerevisiae to weak acids present in lignocellulosic hydrolysate. FEMS Yeast Res..

[45] Nikodinovic-Runic J., Guzik M., Kenny S.T., Babu R., Werker A., O Connor K.E. (2013). Carbon-rich wastes as feedstocks for biodegradable polymer (Polyhydroxyalkanoate) production suing bacteria. Advances in Applied Microbiology.

[46] Arcos-Hernandez M.V., Gurieff N., Pratt S., Magnusson P., Werker A., Vargas A., Lant P. (2010). Rapid quantification of intracellular PHA using infrared spectroscopy: An application in mixed cultures. J. Biotechnol..

[47] Lopes M.S.G., Gosset G., Rocha R.C.S., Gomez J.G.C., Ferreira da Silva L. (2011). PHB biosynthesis in catabolite repression mutant of *Burkholderia sacchari*. Curr. Microbiol..

